# Manifestations and impact of the COVID‐19 pandemic in neuroinflammatory diseases

**DOI:** 10.1002/acn3.51314

**Published:** 2021-02-22

**Authors:** Seth N. Levin, Shruthi Venkatesh, Katie E. Nelson, Yi Li, Ines Aguerre, Wen Zhu, Karman Masown, Kathryn T. Rimmer, Claudiu I. Diaconu, Kaho B. Onomichi, Victoria M. Leavitt, Libby L. Levine, Rebecca Strauss‐Farber, Wendy S. Vargas, Brenda Banwell, Amit Bar‐Or, Joseph R. Berger, Andrew D. Goodman, Erin E. Longbrake, Jiwon Oh, Bianca Weinstock‐Guttman, Kiran T. Thakur, Keith R. Edwards, Claire S. Riley, Zongqi Xia, Philip L. De Jager, Multiple Sclerosis Resilience to COVID‐19 (MSReCOV) Collaborative

**Affiliations:** ^1^ Multiple Sclerosis Center and Center for Translational & Computational Neuroimmunology Department of Neurology and Taub Institute for Research on Alzheimer’s Disease and the Aging Brain Columbia University Irving Medical Center New York New York USA; ^2^ New York Presbyterian Hospital New York New York USA; ^3^ Program in Translational Neuroimmunology Department of Neurology University of Pittsburgh Pittsburgh Pennsylvania USA; ^4^ Department of Neurology Children’s Hospital of Pennsylvania Philadelphia Pennsylvania USA; ^5^ Department of Neurology Perelman School of Medicine University of Pennsylvania Philadelphia Pennsylvania USA; ^6^ Department of Neurology University of Rochester Medical Center, School of Medicine and Dentistry Rochester New York USA; ^7^ Department of Neurology Yale University New Haven Connecticut USA; ^8^ Division of Neurology Department of Medicine St Michael's Hospital University of Toronto Toronto Ontario Canada; ^9^ Department of Neurology Jacobs Multiple Sclerosis Center Jacobs School of Medicine and Biomedical Sciences University at Buffalo State University of New York Buffalo New York USA; ^10^ The Multiple Sclerosis Center of Northeastern New York Latham New York USA

## Abstract

**Objective:**

To report initial results of a planned multicenter year‐long prospective study examining the risk and impact of COVID‐19 among persons with neuroinflammatory disorders (NID), particularly multiple sclerosis (MS).

**Methods:**

In April 2020, we deployed online questionnaires to individuals in their home environment to assess the prevalence and potential risk factors of suspected COVID‐19 in persons with NID (PwNID) and change in their neurological care.

**Results:**

Our cohort included 1115 participants (630 NID, 98% MS; 485 reference) as of 30 April 2020. 202 (18%) participants, residing in areas with high COVID‐19 case prevalence, met the April 2020 CDC symptom criteria for suspected COVID‐19, but only 4% of all participants received testing given testing shortages. Among all participants, those with suspected COVID‐19 were younger, more racially diverse, and reported more depression and liver disease. PwNID had the same rate of suspected COVID‐19 as the reference group. Early changes in disease management included telemedicine visits in 21% and treatment changes in 9% of PwNID. After adjusting for potential confounders, increasing neurological disability was associated with a greater likelihood of suspected COVID‐19 (OR_adj_ = 1.45, 1.17–1.84).

**Interpretations:**

Our study of real‐time, patient‐reported experience during the COVID‐19 pandemic complements physician‐reported MS case registries which capture an excess of severe cases. Overall, PwNID seem to have a risk of suspected COVID‐19 similar to the reference population.

## Introduction

The COVID‐19 pandemic due to SARS‐CoV‐2 poses an unprecedented challenge to persons with multiple sclerosis (MS) and related disorders. We have limited understanding of the risks and impact of COVID‐19 in neuroinflammatory diseases (NID) of the central nervous system, particularly among patients receiving disease‐modifying therapies (DMTs).^1^ The standard of care for NID involves routine in‐person clinic visits, physical assessments, and laboratory and radiological monitoring, which have been abruptly and substantially disrupted during the public health crisis.^2^ COVID‐19 may disproportionally affect persons with NID (PwNID), as these individuals have higher rates of overall infection than the general population and suffer from an increased rate of comorbid health conditions, some of which contribute to COVID‐19 severity.^3^, ^4^

Globally, there is an urgent need to rapidly gather information during the COVID‐19 pandemic to guide the management of PwNID. Available case reports^5^, ^6^ raise intriguing questions, and the physician‐reported national and international COVID‐19 registries^7^, ^8^ will provide key insights. However, these efforts do not sample the majority of the exposed population, as the results are limited to individuals who seek medical attention and generalizable only to the subpopulation with moderate to severe COVID‐19 manifestations. To complement these efforts, we launched a prospective online study to rapidly gather longitudinal, real‐time information over 1 year directly from PwNID and a neurologically asymptomatic reference group. The online design facilitates the survey of a broad population during the pandemic and has the flexibility to capture higher resolution data on selected features. Our multicenter structure enables the collaborative team to leverage the existing clinical and research data infrastructure at each MS Center. Our long‐term research goals are to (1) identify risk factors for the variable manifestations of COVID‐19, (2) assess the impact of the pandemic on patient management as well as on neurological and psychosocial outcomes, and (3) set the stage for downstream biological and clinical evaluations that leverage the longitudinally collected patient‐reported outcomes. Here, we share the baseline results captured during the first peak of the pandemic.

## Methods

### Study design and participants

We recruited adults 18 years or older with NID (MS or a related disorder), as well as a neurologically asymptomatic reference group across the United States to complete online surveys using the Research Electronic Data Capture (REDCap) platform.^9^ Participants were expected to complete each survey within 1 week of receipt. For the initial phase of subject recruitment, we began at the following MS centers with active online research registries of neurologist‐confirmed NID and in regions with high COVID‐19 exposure: Columbia University Irving Medical Center (CUIMC) in New York, NY, and University of Pittsburgh Medical Center (UPMC) in Pittsburgh, PA. In parallel, we deployed the surveys to a reference group, which included control participants in other research studies as well as neurologically asymptomatic participants in a prospective nationwide study of first‐degree family members of MS patients (Genes and Environment in Multiple Sclerosis, GEMS).^10^ To expand recruitment of both PwNID and the general community, we advertised our study using the following platforms: local institution website (CUIMC: https://tinyurl.com/y82eens7; University of Pittsburgh: https://tinyurl.com/y95dxrdt; MS Center newsletters, patient advocacy groups such as the National Multiple Sclerosis Society, social media, and direct outreach to both regional and academic MS centers. We excluded non‐English speakers since several surveys have not been validated beyond English. We confirmed demographic and clinical histories from research registries at each MS center through medical record review. The institutional review boards of the University of Pittsburgh and CUIMC approved the study protocols. All participants provided electronic consent.

### Measurements

We designed the surveys to broadly assess potential risk factors, COVID‐19 status and symptoms, self‐reported neurological and psychosocial outcomes, and changes in neurological care. The direct link to the study surveys is available on REDCap: (1) https://web.neuro.columbia.edu/redcap/surveys/?s=8MJNFDXE4F, or (2) https://redcap.link/covid19‐and‐ms.

#### Potential risk factors

We collected demographic information (age, sex, race, ethnicity, zip code, body mass index [BMI]), smoking behavior (quantity and duration of cigarette and electronic cigarette consumption), comorbidities (adapted from the Charlson Comorbidity Index, and hypertension), and precautionary measures taken in response to the pandemic (handwashing, self‐quarantine, social distancing).

#### COVID‐19 status and symptoms

We quantified the frequency of common COVID‐19 related symptoms, COVID‐19 testing (access and results), and healthcare utilization (emergency room visits and hospitalizations). Using the Centers for Disease Control and Prevention (CDC) guidelines as of April 2020 (https://www.cdc.gov/coronavirus/2019‐ncov/symptoms‐testing/symptoms.html), we defined suspected COVID‐19 as having cough or shortness of breath or any two the following: fever, sore throat, muscle aches, or new loss of taste/smell.

#### Neurological history and change in neurological care

For PwNID, we ascertained diagnosis, disease duration, current, and past DMT as well as any change in management (neurological visits, MRIs, treatments) during the pandemic.

#### Neurological outcomes

We assessed neurological function using three self‐reported outcomes: one general and two disease‐specific. First, we quantified physical function using the National Institute of Health Patient‐Reported Outcomes Measurement Information System (PROMIS) Physical Function (version 1.2). PROMIS is a nationally validated, computer adaptive test to measure self‐reported health in patients across a range of chronic diseases and demographics.^11^ T‐scores from the US general population have a normal distribution with a mean score of 50 and a standard deviation of 10. We chose this outcome to detect differences in physical function among PwNID and the reference group and to longitudinally differentiate clinically meaningful changes. Second, we measured neurological function using the Multiple Sclerosis Rating Scale‐Revised (MSRS‐R), assessing eight self‐reported neurological domains (walking, upper limb function, vision, speech, swallowing, cognition, sensory, bladder, and bowel function; each domain is scored as 0–4, with 4 indicating severe disability). MSRS‐R correlates with clinical measures of disability and is validated for MS.^12^, ^13^ Finally, we evaluated self‐reported gait impairment using Patient Determined Disease Steps (PDDS). The PDDS is an ordinal scale from 0 to 8, with 0 indicating no impairment and 8 representing bed‐bound status. The PDDS approximates rater‐performed metrics of neurological function such as the Extended Disability Status Scale.^14^ While MSRS‐R and PDDS are correlated, the former provides more granular subscales and the latter better defines gait impairment.^13^

#### Psychosocial outcomes

Given the broad social impact of COVID‐19, we collected standardized self‐assessments of mood, social support, and loneliness. For mood, we utilized the PROMIS Depression (version 1.0) scale, in which higher T‐scores correlate with greater depressive symptoms. PROMIS has higher internal consistency than other measures of mood in MS^15^ and facilitates measurement of changes in depression severity over time. We characterized depression severity using previously defined thresholds.^16^ For social support, we utilized the Modified Social Support Survey 5‐item (MSSS‐5).^17^ After converting scores (0–100, with higher scores indicating greater perceived support), we dichotomized scores based on the 10^th^ percentile (≤40) to characterize the level of support. Finally, we quantified the present state of loneliness as the total score (20–80 with higher scores indicating greater loneliness) on the UCLA Loneliness Scale, a 20‐item measure previously used in MS.^18^

#### Additional questions

Mask and glove wearing by the participant and household members, lung imaging, and pregnancy status will be reported in future reports, as these questions were incorporated after the deployment of the baseline survey due to changing guidelines.

### Map

We extracted confirmed COVID‐19 cases and population size estimates as of 1 May 2020 from the Johns Hopkins University COVID Resource Center (https://github.com/CSSEGISandData/COVID‐19). We mapped the case prevalence of COVID‐19 per 100,000 for each county in the United States in a heat map and superimposed study participants based on the zip code of their residence using R (version 3.5.0).

### Statistical analyses

We compared the demographic and clinical characteristics of the PwNID and reference groups using (1) t‐tests for age, BMI, PROMIS Physical function T‐score, PROMIS Depression T‐Score, Loneliness Score, and MSSS‐5, (2) chi‐squared or Fisher’s exact tests for dichotomous variables of sex, race, and proportion of persons with specific symptoms or health behaviors, (3) nonparametric Wilcoxon rank‐sum tests for Charlson Comorbidity Index (CCI).

We performed multivariate logistic regressions to measure the contribution of NID or neurological disability to depression severity, low perceived social support, and suspected COVID‐19 after adjusting for clinically significant confounders. Analyses were completed using SPSS version 25.0 (IBM Corp., Armonk, NY).

## Results

### Baseline characteristics of subjects and potential risk factors

**Figure 1:**
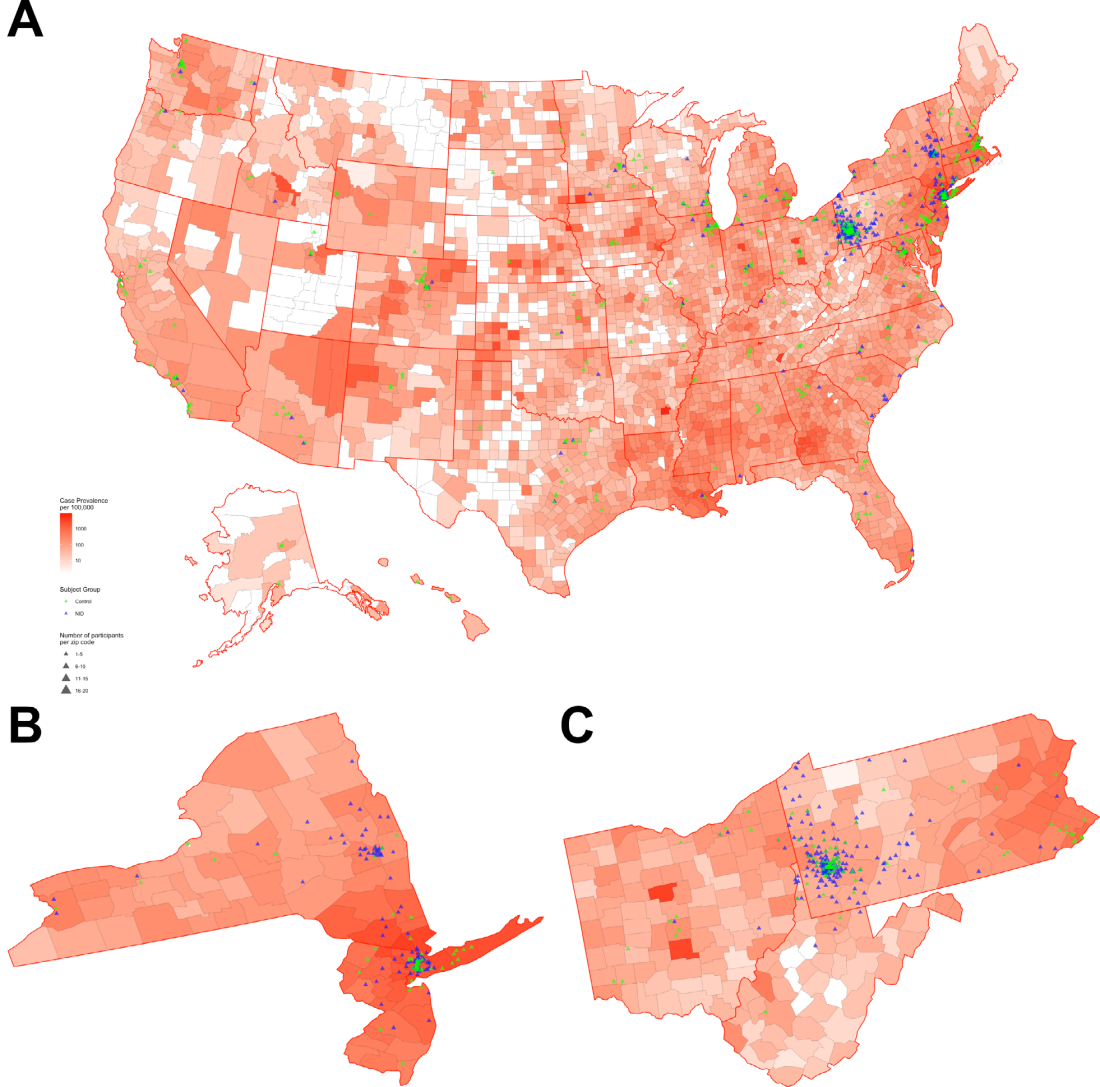
The Multiple Sclerosis Resilience to COVID‐19 (MSReCOV) Study: scope of enrollment. The map illustrates the county‐level variation in case prevalence of COVID‐19 per 100,000 across the United States as of 1 May 2020. Persons with neuroinflammatory disease (NID) are shown as blue triangles and the reference participants are depicted as green triangles. The size of the triangle indicates the number of participants per zip code. (A) Entire United States. (B) New York and New Jersey. (C) Pennsylvania, Ohio and West Virginia.

Study recruitment began in early April 2020, and a data freeze for this analysis occurred on 30 April 2020. Of the invited 4715 participants from institutional research registries, 841 (18%) consented to the study. An additional 274 individuals enrolled through the public survey link. We conducted the initial analysis on 1115 participants, predominantly representing states and counties with high but diverse case prevalence of COVID‐19 (Fig. 1). Six hundred and thirty (57%) participants have MS or a related NID, whereas 485 (43%) represent reference adults, including 251 control participants from the community and 234 neurologically asymptomatic participants with first‐degree family history of MS^10^ (Table 1). The NID group is older (50.0 ± 12.1 vs. 43.3 ± 11.3 years, *P* < 0.001), includes more women (83% vs. 76%, *P* = 0.009), has more comorbidities (CCI: 1 [0, 2] vs. 0 [0, 1], *P* < 0.001), has a higher (BMI: 28.5 ± 7.5 vs. 26.7 ± 6.8, *P* = 0.001), has lower general physical function (PROMIS T‐score: 45.1 ± 11.1 vs. 57.7 ± 8.8, *P* < 0.001), and includes more current or past smokers (34% vs. 20%, *P* < 0.001) than the reference group. The Northeastern United States is the most represented region of residence among surveyed participants, particularly for the NID group (*n* = 533, 87% of PwNID).

**Table 1:** Baseline demographic and clinical characteristics.

Characteristic	Reference (*N* = 485)	Neuroinflammatory disorder (*N* = 630)	*P* value
Age, mean (SD) years	43.3 (11.3)	50.0 (12.1)	<0.001
Female sex, *N* (%)	368 (76)	520 (83)	0.009
Race, *N* (%)			
Caucasian	461 (95)	586 (93)	
African American	6 (1)	22 (3.5)	
Asian	4 (1)	4 (0.5)	0.159^1^
Multiracial	8 (2)	7 (1)	
Other	6 (1)	11 (2)	
Hispanic, *N* (%)	15 (3)	21 (3)	0.934
Geographic region, *N* (%)			
Northeast	190 (41)	533 (87)	
Midwest	95 (21)	29 (5)	<0.001
South	92 (20)	39 (6)	
West	84 (18)	15 (2)	
CCI, median [IQR]	0 [0,1]	1 [0,2]	<0.001
BMI, mean (SD)^2^	26.7 (6.8)	28.5 (7.5)	0.001
Ever smoker, *N* (%)	99 (20)	215 (34)	<0.001
Current smoker, *N* (%)	12 (3)	49 (8)	<0.001
PROMIS physical T‐score, mean (SD)	57.7 (8.8)	45.1 (11.1)	<0.001
PROMIS depression T‐score, mean (SD)	51.3 (7.5)	52.2 (8.5)	0.088
Depression severity, *N* (%)			
Mild	135 (29)	142 (24)	
Moderate	63 (13)	106 (18)	0.001^3^
Moderately severe	7 (2)	27 (5)	
Severe	4 (1)	11 (2)	
MSSS‐5, mean (SD)	79.1 (22.3)	75.6 (25.1)	0.017
Loneliness score, mean (SD)^4^	39.7 (10.0)	39.5 (11.8)	0.404
Increased handwashing, *N* (%)	466 (97)	622 (94)	0.033
Handwashing frequency, *N* (%)			
≤3 times per day	25 (5)	40 (6)	
4–6 times per day	127 (26)	146 (23)	0.017^5^
7–9 times per day	149 (31)	157 (25)	
≥10 times per day	183 (38)	380 (45)	
Social distancing adherence, *N* (%)	473 (98)	606 (98)	0.877
Household social distancing adherence, *N* (%)	441 (92)	574 (92)	0.633
Self‐quarantine, *N* (%)	57 (12)	100 (16)	0.043

CCI, Charlson Comorbidity Index, BMI, body mass index; PROMIS, Patient‐Reported Outcomes Measurement Information System; MSSS‐5, Modified Social Support Survey 5‐item.

^1^ Caucasian versus Non‐Caucasian.

^2^ Subgroup of 805 participants with body mass index (BMI) data.

^3^ Depression severity was defined using established thresholds^17^: Mild (52.5–58.6), Moderate 58.7–64.7), moderately severe (64.8–70.3), and severe (>70.3). Statistical analysis based on moderate to severe versus mild depression.

^4^ UCLA loneliness.

^5^ Handwashing >10 versus <10 times per day.

We next assessed precautionary behavior in response to the COVID‐19 pandemic. Both groups endorsed a high daily frequency of hand washing and a high personal and household adherence to social distancing. PwNID were more likely to wash hands ≥10 times daily and to have self‐quarantined themselves than reference participants (Table 1).

### Change in neurological care

To capture the impact of the pandemic on the broader NID population, we analyzed the MS (*n* = 614, 97.5%) and related disorders (*n* = 16, 2.5%) together (Table 2). The mean disease duration was 18.1 (11.7) years, and there was a mild‐to‐moderate burden of disability, with a median PDDS of 1 [0, 4] and a median MSRS‐R of 7 [3, 11]. Usage of DMT during the pandemic was common (*n* = 553, 86%), including injectable (*n* = 116, 19%), oral (*n* = 104, 17%), and infusion (*n* = 314, 50%) options (Table 2). Notably, B‐cell depleting monoclonal antibody therapies (rituximab, ocrelizumab) were the most common DMTs among PwMS (36%) and among those with related disorders (*n* = 7, 43%). Given the self‐reported nature of the study, we verified key elements, including accuracy of NID diagnosis and DMT usage, in a subset of 150 participants who are enrolled in CUIMC and UPMC research registries and found 99% concordance between recorded and self‐reported information.

**Table 2:** Characteristics of neuroinflammatory disease group and change in care.

Characteristic	*N* = 630
Diagnosis, *N* (%)	
Multiple sclerosis	614 (98)
Neuromyelitis optica spectrum disorder	8 (1)
Isolated transverse myelitis	4 (<1)
Isolated optic neuritis	3 (<1)
Neurosarcoidosis	1 (<1)
Disease duration, mean (SD) years	18.1 (11.7)
MSRS‐R, median [IQR]	7 [3, 11]
PDDS, median [IQR]	1 [0, 4]
Current DMT, *N* (%)^1^	
None	87 (14)
Injectable	116 (19)
Oral	104 (17)
Infusion	314 (50)
Prior DMT, *N* (%)	415 (66)
Relapse in previous 2 weeks, *N* (%)	45 (7)
Change in neurology clinic visit due to COVID‐19, *N* (%)	
No change	422 (68)
Telemedicine visit	129 (21)
Cancelled or postponed	65 (11)
Change in DMT due to COVID‐19, *N* (%)	
No change	560 (91)
Stopped DMT	15 (2)
Reduced DMT frequency	43 (7)

MSRS‐R, Multiple Sclerosis Rating Scale‐Revised; PDDS, Patient Determined Disease Steps; DMT, disease‐modifying therapy.

^1^ Disease‐Modifying Therapy; see Table S4 for the complete list.

We further assessed the impact of the COVID‐19 pandemic on neurological care (Table 2). In the short period since the onset of the pandemic, a substantial subgroup (*n* = 194, 32%) reported a change in clinical encounters: Their neurologist either switched to telemedicine visits (21%) or canceled or postponed their visit (11%). A few participants (*n* = 58, 9%) reported changes in DMT due to COVID‐19, including stopping DMT (*n* = 15, 2%) or decreasing DMT frequency (*n* = 43, 7%). During the study period, self‐reported neurological relapses, defined as new neurological events lasting persistently longer than 24 hours, occurred in 45 (7%) participants.

### Psychosocial factors

Based on surveys conducted at the peak of the pandemic, depression rates were similar between the two groups, but more PwNID had moderate to severe depression symptoms than the reference population. Perceived social support (MSSS‐5) was lower among PwNID (75.6 ± 25.1 vs. 79.1 ± 22.3, *P* = 0.017), but there was no difference in self‐reported loneliness (Table 1). After adjusting for potential confounders (age, sex, race/ethnicity, comorbidity, social support in analysis of depression, and depression in analysis of social support), having NID increased one’s odds of moderate‐to‐severe depression (OR_adj_ = 2.22, 1.48–3.31, *P* < 0.001) and of lower social support (OR_adj_ = 1.82, 1.17–2.83, *P* = 0.008) (Table 3, Table S1).

**Table 3:** Multivariable analyses of outcomes.

Independent factor	Outcome	OR	95% CI
NID	Moderate to severe depression^1^	2.22*	1.48–3.31
NID	Low social support^2^	1.82**	1.17–2.83
MSRS‐R^3^	Suspected COVID‐19^4^	1.45**	1.11–1.78

NID, neuroinflammatory disorders; MSRS‐R, Multiple Sclerosis Rating Scale‐Revised; CCI, Charlson Comorbidity Index.

^1^ Moderate to severe depression defined as PROMIS depression T‐score >58.6 using established thresholds.^17^ The reference is mild depression (52.5–58.6). Logistic regression analyses for severe depression were adjusted for the pertinent covariates: age, sex, race, CCI, and low social support. See Table S1 for full model.

^2^ Low social support defined as modified social support survey‐5 (MSSS‐5) item converted score of <40 based on 10^th^ percentile threshold. The reference was MSSS‐5 score >40. Logistic regression analyses for low social support were adjusted for the pertinent covariates: age, sex, race, CCI, and depression. See Table S1 for the full model.

^3^ Multiple Sclerosis Rating Scale revised; MSRS‐R raw scores were converted to z‐scores. The OR reflects a one standard deviation change in MSRS‐R value.

^4^ Suspected COVID‐19 cases based on CDC symptom criteria, including cough or shortness of breath OR any two of the following: fever, muscle pain, sore throat, new loss of taste or smell. Logistic regression analysis of suspected COVID‐19 was adjusted for pertinent covariates: age, sex, race, CCI, depression, smoking status, disease‐modifying therapy, and state residency. See Table S1 for the full model.

* *P* < 0.001.

** *P* < 0.01.

### COVID‐19 status, symptoms and healthcare access

Given the low COVID‐19 testing rate in the United States around the time of baseline survey completion for this study (April 2020), we chose to focus on potential symptoms of COVID‐19, using the CDC criteria (https://www.cdc.gov/coronavirus/2019‐ncov/symptoms‐testing/symptoms.html) at the launch of the study to characterize suspected cases (Table 4). A subset (*n* = 204, 18%) of the study cohort met the criteria for suspected COVID‐19. Overall, both NID and reference groups experienced similar frequencies of COVID‐19 symptoms.

**Table 4:** Suspected COVID‐19 and healthcare utilization.

Characteristic	All (*N* = 1115)	Reference (*N* = 485)	Neuroinflammatory disorder (*N* = 630)	OR	95% CI
Suspected COVID‐19, *N* (% total)^1^	202 (18)	98 (20)	104 (17)	0.78	0.56–1.06
Symptom type, *N* (% suspected)					
Fever	68 (34)	33 (34)	33 (34)	1.00	0.56–1.79
Cough	164 (81)	79 (81)	85 (82)	1.08	0.53–2.18
Shortness of breath	60 (30)	32 (33)	28 (27)	0.76	0.42–1.40
Sore throat	108 (53)	52 (53)	56 (54)	1.03	0.59–1.80
Muscle aches	88 (44)	40 (40)	48 (46)	1.24	0.71–2.17
Loss of taste or smell	30 (15)	15 (15)	15 (14)	0.93	0.43–2.03
Any testing for COVID‐19, *N* (% suspected)	44 (22)	20 (20)	24 (23)	0.93	0.51–1.70
Tested positive for COVID‐19, *N* (% tested)	13 (31)	6 (30)	7 (29)	1.02	0.28–3.77
Reasons for no testing, *N* (% suspected)					
Did not seek testing^2^	88 (56)	45 (54)	43 (49)	0.83	0.46–1.51
Not offered testing	33 (21)	20 (24)	13 (16)		
No access to testing	10 (6)	4 (5)	6 (7)		
Sought guidance from healthcare professional, *N* (% total)	170 (15)	52 (11)	118 (19)	1.94*	1.37–2.76
Visited an urgent care or emergency room, *N* (% total)	56 (5)	17 (4)	39 (6)	1.83**	1.02–3.28
Hospitalized, *N* (% total)	12 (1)	3 (1)	9 (2)	2.36	0.64–8.77

^1^ Based on CDC symptom criteria, including cough or shortness of breath OR any two of the following: fever, muscle pain, sore throat, new loss of taste or smell.

^2^ Did not seek testing versus other.

* *P* < 0.01.

** *P* < 0.05.

In the overall cohort, few (*n* = 44, 4%) had undergone COVID‐19 testing, and 13 of those tested positive. Of the suspected cases who did not undergo testing, 88 (51%) did not seek testing, 33 (20%) were not offered testing by a healthcare professional, and 10 (6%) reported no access to testing. There was no difference in testing rate between PwNID and reference participants. In the month preceding the baseline survey, PwNID required more urgent or emergency care (6% vs. 4%, *P* = 0.039) and sought more advice from healthcare professionals (19% vs. 11%, *P* = 0.001), but did not have a higher rate of hospitalizations (2% vs 1%, *P* = 0.186) than the reference group (Table 4).

### Impact of comorbidities and treatment on suspected COVID‐19

After adjusting for potential confounders (age, sex, race/ethnicity, comorbidity, depression, smoking status, residency, DMT status), increasing neurological disability (based on MSRS‐R) was associated with a greater likelihood of suspected COVID‐19 (OR_adj_ = 1.45, 1.17–1.84, *P* = 0.001) (Table 3, Table S1). In exploratory analyses, we examined the role of potential risk factors and disease‐modifying treatments in suspected COVID‐19 in this cohort (see Supplementary Material, File S2, Tables S2–S4).

## Discussion

We report the launch of the MSReCOV study, a longitudinal investigation of the risk and impact of COVID‐19 in NID. Leveraging a collaborative network of MS centers, we report the baseline assessment of the first 1115 adult participants enrolled during the height of the initial phase of the COVID‐19 pandemic in North America, from 3 April to 30 April. Many of our participants reside in areas with the highest known prevalence of COVID‐19.

PwNID had a higher comorbidity burden and greater physical impairment than the reference population. In these regards, our study population is similar to other registries in North America.^19^, ^20^ Based on the catchment areas of the participating MS centers, we are sampling PwNID and reference participants from diverse environments, including urban, suburban, and rural locales. While there is some representation from racial and ethnic minorities, they are under‐represented in the first phase of our enrollment. We will commit efforts to increase minority enrollment by reaching out to local advocacy organizations and through targeted study advertisements.

Since information with which to guide clinical practice of MS and other NID during the pandemic is limited, there is an urgent need for robust data. Our study provides early insights based on the patient experience that complements data from physician‐reported case series with the caveat of reliance on self‐reported symptoms. We were also constrained by extremely limited testing for COVID‐19 in the United States, with none but the most severely affected individuals tested during this period that ended in April 2020. Nevertheless, our analyses returned several insights. PwNID report more severe depression and lower perceived social support. Increasing sample size and longitudinal data collection will enable us to examine these associations and other questions in detail, including temporal trends and the important issue of causality among the associated factors.

Although the overall rates of suspected COVID‐19 and positive COVID‐19 test results are similar between PwNID and reference participants in this cohort, we need further research, as the NID population is heterogeneous and neurological disability has been reported to increase infection risk in other contexts.^21^, ^22^ Indeed, neurological disability is associated with suspected COVID‐19 in our study. Persons with MS require more healthcare resources than the general population, including emergency room visits and hospitalizations,^23^ often due to relapse or pseudo‐relapse in the setting of infection.^24^ Here, PwNID with suspected COVID‐19 sought more urgent or emergency care, though we found no statistically significant increase in hospitalizations or relapses.

Reassuringly, we observe similarly high adherence to public health recommendations of precautionary measures in both NID and reference groups. A central part of the recommendation is social distancing.^25^ However, the precipitous change in social dynamics due to the pandemic response may disproportionally affect PwNID in terms of receiving home care, accessing healthcare resources, and experiencing life satisfaction. Severe depression and low perceived support were more prevalent in the NID group. Although we did not observe a baseline difference in loneliness scores, loneliness may increase as the pandemic persists.^26^ Loneliness is an independent determinant of health,^27^ and influences quality of life in MS.^28^ Consequently, beyond COVID‐19, clinicians should address the growing emotional hardships affecting PwNID.

In response to COVID‐19, the standard of care has rapidly changed for many patients. While 11% of the PwNID reported canceled or postponed neurology visits, 21% reported virtual telemedicine visits. Whether a telemedicine visit is equivalent to a conventional in‐person visit likely depends on a number of factors such as disease status and patient–physician relationship. Previous studies showed that telemedicine is feasible, cost‐effective, and well‐received by patients.^29^, ^30^ As telemedicine visits increase, clinicians may need to incorporate and expand other forms of assessment into their decision making, including wearable technologies, smartphone‐based‐applications, and patient‐reported outcome measures.^31^

The relationship of DMTs and COVID‐19 is of interest. Although our study is not yet well powered to compare differences in suspected COVID‐19 across DMTs, the initial results provide important insights, with all but one treatment showing no difference in association with suspected COVID‐19 (Table S4). Approximately 9% PwNID stopped DMT or reduced frequency due to COVID‐19. While our surveys did not capture the rationale for these changes, possibilities include response to infection or perceived risks of treatment. Delays in infusions could also have developed as clinics and hospitals restricted nonessential procedures. The potential interruptions in DMT management and the methods to address these issues require further investigation.

A major strength of this multicenter study design is that it leverages existing and ongoing research efforts at each site to enable future biological studies. In this first report from MSReCoV, we presented the baseline results by aggregating data from the early participating MS centers. We expect to perform replication as the initial sites scale up recruitment and as additional collaborating MS centers begin enrollment. To facilitate collaboration and integration with other national and international research efforts, we have shared the study design with a growing number of potential collaborators, and key questionnaires are available through the REDCap shared library to facilitate repurposing by other groups.

The self‐reported nature of our study design is an important limitation to generalization. The restrictions on in‐person research and clinical activities at the launch of the study mandated alternative methods of data collection such as online questionnaires. For the main MS disability measures, we chose two scales based on prior studies that showed overall consistency with neurological examinations: MSRS‐R and PDDS.^13^, ^14^ We also included the PROMIS physical function scale developed by the National Institutes of Health as a generalizable measure of function across health and disease.^32^ Mirroring the general population facing the limited testing availability in this phase of the pandemic, our cohort has low COVID‐19 testing rates.^33^ Consequently, we cannot directly confirm COVID‐19 at this time and instead use the April 2020 CDC criteria for suspected COVID‐19 as a reasonable proxy. Interestingly, self‐reported symptoms have a reasonable accuracy (area under the curve = 0.76) in predicting COVID‐19 in the general population.^34^ We will perform a medical record review of confirmed or suspected COVID‐19 cases for future validation as testing expands. Furthermore, our participants are most likely skewed toward persons with no or mild COVID‐19 symptoms, as the more severely affected individuals would be less inclined to engage in survey studies. Therefore, our study population complements the patient population in case registries who likely have moderate to severe manifestations of COVID‐19. Importantly, patient‐reported measures provide unique and complementary information regarding health behaviors, patient‐centered outcomes, and the psychosocial impact of the pandemic.

Our study has other limitations. First, the online survey format has inherent selection bias as it does not effectively reach populations such as minorities, the elderly, the economically disadvantaged with limited access to the internet, non‐English speakers, or those who were unable to complete the online questionnaire due to cognitive impairment. Furthermore, comorbid depression could have influenced study participation and self‐report of suspected COVID‐19. Second, reference participants differ from PwNID with respect to demographic characteristics, including age and sex. Older age in the NID group could contribute to the higher comorbidities and smoking frequency. We adjusted for these variables in multivariable analyses of NID in association with depression and social support. These differences do not affect analyses of traits among PwNID. Third, the NID population includes a higher percentage of individuals receiving DMT compared to other North American registries,^35^ which could impact the generalizability of our findings. We adjusted for DMT use in multivariable analysis of suspected COVID‐19. Finally, the reference population represents a mixture of recruitment sources, with an important contribution from the (GEMS) study,^10^ which consists of neurological asymptomatic first‐degree relatives of MS patients. The asymptomatic GEMS participants could have health behaviors and other profiles that differ from the general population, but we saw no difference in their rates of suspected COVID‐19 when compared with other reference participants (18% vs. 22%, *P* = 0.332). As the study expands, we will continue to recruit reference participants from the broader community.

Our collaborative multicenter study has rapidly created a large cohort that we plan to examine longitudinally and to integrate with research data from each center. This cohort study forms a robust foundation for biological sampling that will enable the investigation of host susceptibility, immunological response (e.g., to a future SARS‐CoV‐2 vaccine, development of SARS‐CoV‐2 antibody), and other biomarkers relating to DMTs and COVID‐19. Our baseline data highlight the early impact of COVID‐19. As the pandemic evolves, acquiring time‐sensitive, real‐world data will critically guide individualized management of PwNID in a dynamic manner, particularly in assisting those at high risk from the physical and psychosocial consequences of COVID‐19.

## Conflict of Interest

Dr. Levin has received a fellowship training grant through Genentech and honoraria for advisory work with Biogen, which manufacture medications reported in this study. Dr. Vargas has received honoraria for advising or consulting work with Biogen, Alexion, EMD Serono, and grant support from Teva, which manufacture medications reported in the study. Dr. Banwell serves on the scientific advisory board for Biogen, Sanofi, Novartis, and has received honoraria for advising or consulting work with Novartis, which manufacture medications reported in this study. Dr. Edwards has received speaking and consulting fees from Biogen and Genzyme and research support from Biogen, Genentech, Novartis, and Sanofi, which manufacture medications reported in the study. Dr. Longbrake has received honoraria for advisory or consulting work with Biogen, Celegene/Bristol Myers Squibb, Genentech, Genzyme, EMD Serono, which manufacture medications reported in this study. Dr. Weinstock‐Guttman has participated in speaker's bureaus, received grant support, and/or served as a consultant for Biogen, EMD Serono, Novartis, Genentech, Celgene, which manufacture medications reported in this study. Dr. Bar‐Or serves on scientific advisory boards for Biogen, Celgene, EMD Serono, Novartis, Roche/Genentech and Sanofi‐Genzyme, and has sponsored research agreements with Biogen, Novartis and Roche/Genentech, which manufacture medications reported in this study. Dr. Berger serves on the scientific advisory board of Novartis, has received honoraria for advisory work with Biogen, Genentech/Roche, Celgene, Novartis, Genzyme, EMD Serono, and has received institutional grant support from Biogen and Genentech/Roche, which manufacture medications reported in the study. Dr. Goodman has received research support from Biogen, Genentech/Roche, Sanofi Genzyme, and Teva, which manufacture medications reported in this study. Dr. Oh has received grants and personal fees from Biogen, Roche, and Sanofi Genzyme, and personal fees from Celgene, Novartis, and EMD Serono, which manufacture medications reported in this study. Dr. Riley has received honoraria for advisory or consulting work with Biogen, Genentech/Roche, EMD Serono, Celgene, Teva, and Genzyme, which manufacture medications reported in this study. Dr. Xia serves on the scientific advisory board of Roche/Genentech, which manufactures medications reported in this study. Dr. De Jager serves on the scientific advisory board for Roche, Biogen, Celgene, has a sponsored research agreement with Biogen and Roche, and has fellowship funding through Genentech, which manufacture medications reported in this study.

## Author Contributions

See File S1 for information about the study group members and affiliations.

## Supporting information


**File S1.** A full list of MSReCOV members and author contributions.

**File S2.** Exploratory analyses examining risk factors and disease‐modifying treatments associated with suspected COVID‐19 in people with neuroinflammatory disorders (NID).
**Table S1.** Multivariable Analyses of Depression and Social Support, and Suspected COVID‐19 symptoms.
**Table S2.** Factors associated with suspected COVID‐19 in the entire study population.
**Table S3.** Factors associated with suspected COVID‐19 in the neuroinflammatory group.
**Table S4.** Current disease modifying therapy among the participants with neuroinflammatory disorders in relation to CDC criteria for suspected COVID‐19.
