# Supplementary material for: Manifestations and impact of the COVID‐19 pandemic in neuroinflammatory diseases
Source: Ann Clin Transl Neurol. 2021 Feb 22;8(4):918–28. doi: 10.1002/acn3.51314 (PMC8013889; doi:10.1002/acn3.51314)
Supplement: Supplementary file 1 — File S1. A full list of MSReCOV members and author contributions. [file ACN3-8-918-s001.docx]

**Supplemental File 1.**

**Members of the Multiple Sclerosis Resilience to COVID-19 (MSReCOV) Collaborative**

| **Name** | **Institution** |
| --- | --- |
| Ines Aguerre, BSc | Columbia University Irving Medical Center |
| Brenda Banwell, MD | Children’s Hospital of Pennsylvania |
| Amit Bar-Or, MD | University of Pennsylvania School of Medicine |
| Joseph Berger, MD | University of Pennsylvania School of Medicine |
| Philip De Jager, MD, PhD | Columbia University Irving Medical Center |
| Megan Dahl, BSc | University of Pittsburgh Medical Center |
| Claudiu Diaconu, MD, MSc | Columbia University Irving Medical Center |
| Fatoumata Diallo, BSc | Columbia University Irving Medical Center |
| Keith R. Edwards, MD | The Multiple Sclerosis Center of Northeastern NY |
| Samantha Epstein, MD | Columbia University Irving Medical Center |
| Andrew Goodman, MD | University of Rochester Medical School |
| Melanie Guenette, MSc | St. Michael’s Hospital, University of Toronto |
| Vineetha Kamath, BSc | The Multiple Sclerosis Center of Northeastern NY |
| Katelyn Kavak, MSc | Jacobs School of Medicine and Biomedical Sciences |
| Anne Kever, PhD | Columbia University Irving Medical Center |
| Victoria Leavitt, PhD | Columbia University Irving Medical Center |
| Seth Levin, MD | Columbia University Irving Medical Center |
| Libby Levine, ANP-BC | Columbia University Irving Medical Center |
| Elle Levit, MD | Yale University School of Medicine |
| Yi Li, MD | University of Pittsburgh Medical Center |
| Erin Longbrake, MD PhD | Yale University School of Medicine |
| Fred Lublin, MD | Icahn School of Medicine |
| Karman Masown, BSc | University of Pittsburgh Medical Center |
| Cornelius Muntazar, BSc | Columbia University Irving Medical Center |
| Nancy Nealon, MD | Weill Cornell Medical Center |
| Katherine Nelson, BSc | Columbia University Irving Medical Center |
| Jiwon Oh, MD, PhD | St. Michael’s Hospital, University of Toronto |
| Kaho B. Onomichi, MSc | Columbia University Irving Medical Center |
| Kara Patrick, MSc | Jacobs School of Medicine and Biomedical Sciences |
| Christopher Perrone, MD | University of Pennsylvania School of Medicine |
| Claire Riley, MD | Columbia University Irving Medical Center |
| Kathryn Rimmer, MD | Columbia University Irving Medical Center |
| Rosila Sarrosa, MT, AMT | The Multiple Sclerosis Center of Northeastern NY |
| Rebecca Straus Farber, MD | Columbia University Irving Medical Center |
| Kiran Thakur, MD | Columbia University Irving Medical Center |
| Wendy Vargas, MD | Columbia University Irving Medical Center |
| Timothy Vartanian, MD PhD | Weill Cornell Medical Center |
| Caila Vaughn, PhD, MPH | Jacobs School of Medicine and Biomedical Sciences |
| Shruthi Venkatesh, BSc | University of Pittsburgh Medical Center |
| Libby Walker, BSc | University of Pittsburgh Medical Center |
| Bianca Weinstock-Guttman, MD | Jacobs School of Medicine and Biomedical Sciences |
| Sarah Wesley, MD | Columbia University Irving Medical Center |
| Zongqi Xia, MD PhD | University of Pittsburgh Medical Center |
| Wen Zhu, MD MS | University of Pittsburgh Medical Center |

**Author Contributions**

| **Name** | **Location** | **Role** | **Contribution** |
| --- | --- | --- | --- |
| Seth Levin, MD | Columbia University Irving Medical Center, New York City | Author | Design and conceptualization of the study; data collection and analysis; drafting the manuscript |
| Shruthi Venkatesh, BS | University of Pittsburgh, Pittsburgh | Author | Design and conceptualization of the study; data collection and analysis; drafting the manuscript |
| Katie Nelson, BS | Columbia University Irving Medical Center, New York City | Author | Design and conceptualization of the study; data collection and analysis |
| Yi Li, MD | University of Pittsburgh, Pittsburgh | Author | Design and conceptualization of the study; data collection |
| Ines Aguerre, BS | Columbia University Irving Medical Center, New York City | Author | Design and conceptualization of the study; data collection |
| Wen Zhu, MD, MS | University of Pittsburgh, Pittsburgh | Author | Data collection |
| Karman Masown, BS | University of Pittsburgh, Pittsburgh | Author | Data collection |
| Kathryn Rimmer, MD | Columbia University Irving Medical Center, New York City | Author | Data collection |
| Claudiu Diaconu, MD, MS | Columbia University Irving Medical Center, New York City | Author | Data collection |
| Kaho Onomichi, MS | Columbia University Irving Medical Center, New York City | Author | Data collection |
| Victoria Leavitt, PhD | Columbia University Irving Medical Center, New York City | Author | Data collection; Drafting the manuscript |
| Libby Levine, RN, ANP-BC | Columbia University Irving Medical Center, New York City | Author | Data collection; Drafting the manuscript |
| Rebecca Farber, MD | Columbia University Irving Medical Center, New York City | Author | Data collection; Drafting the manuscript |
| Wendy Vargas, MD | Columbia University Irving Medical Center, New York City | Author | Data collection; Drafting the manuscript |
| Brenda Bandwell, MD | Children’s Hospital of Pennsylvania, Philadelphia | Author | Data collection; Drafting the manuscript |
| Amit Bar-Or, MD | University of Pennsylvania, Philadelphia | Author | Data collection; Drafting the manuscript |
| Joseph Berger, MD | University of Pennsylvania, Philadelphia | Author | Data collection; Drafting the manuscript |
| Erin Longbrake, MD, PhD | Yale University, New Haven | Author | Data collection; Drafting the manuscript |
| Andrew Goodman, MD | University of Rochester Medical Center, Rochester | Author | Data collection; Drafting the manuscript |
| Jiwon Oh, MD, PhD | University of Toronto, Toronto | Author | Data collection; Drafting the manuscript |
| Bianca Weinstock-Guttman, MD | State University of New York at Buffalo, Buffalo | Author | Data collection; Drafting the manuscript |
| Keith Edwards, MD | Multiple Sclerosis Center of Northeastern New York, Latham | Author | Data collection; Drafting the manuscript |
| Kiran Thakur, MD | Columbia University Irving Medical Center, New York City | Author | Data collection; Drafting the manuscript |
| Claire Riley, MD | Columbia University Irving Medical Center, New York City | Author | Design and conceptualization of the study; Drafting the manuscript |
| Zongqi Xia, MD, PhD | University of Pittsburgh, Pittsburgh | Author | Design and conceptualization of the study; Drafting the manuscript |
| Philip De Jager, MD, PhD | Columbia University Irving Medical Center, New York City | Author | Design and conceptualization of the study; Drafting the manuscript |
