# Supplementary material for: Manifestations and impact of the COVID‐19 pandemic in neuroinflammatory diseases
Source: Ann Clin Transl Neurol. 2021 Feb 22;8(4):918–28. doi: 10.1002/acn3.51314 (PMC8013889; doi:10.1002/acn3.51314)
Supplement: Supplementary file 2 — File S2. Exploratory analyses examining risk factors and disease‐modifying treatments associated with suspected COVID‐19 in people with neuroinflammatory disorders (NID). Table S1. Multivariable Analyses of Depression and Social Support, and Suspected COVID‐19 symptoms. Table S2. Factors associated with suspected COVID‐19 in the entire study population. Table S3. Factors associated with suspected COVID‐19 in the neuroinflammatory group. Table S4. Current disease modifying therapy among the participants with neuroinflammatory disorders in relation to CDC criteria for suspected COVID‐19. [file ACN3-8-918-s002.docx]

**Supplementary Material**

**Exploratory Analysis on Suspected COVID-19 in Neuroinflammatory Disorders**

In exploratory analyses examining risk factors and disease-modifying treatments associated with suspected COVID-19 in people with neuroinflammatory disorders (NID), we used the CDC criteria for suspected COVID-19 symptoms as of April 2020 (www.cdc.gov/coronavirus/2019-ncov/symptoms-testing/symptoms.html). First, we examined potential risk factors for suspected COVID-19 based on associations reported in the general population (Supplementary Table 2). ^1^ In the overall cohort, participants with suspected COVID-19 (based on the CDC criteria) tended to be younger and were more likely to self-identify as a member of a racial or ethnic minority group. Individuals with symptoms also had a modestly higher proportion of self-reported liver disease (3% vs. 1%, p=0.040) and depression (55% vs. 47%, p=0.044) than those without any potential COVID-19 symptoms.

Among PwNID, participants with suspected COVID-19 had higher rates of depression (n=64, 62% vs. n=247, 47%, p=0.007), greater neurological disability (MSRS-R: 9+5.7 vs. 7+5.4, p<0.001), and more urgent or emergent visits (n=14, 14% vs. n=25, 5%, p=0.001) than those without any suspected COVID-19 symptoms (Supplementary Table 3)**.**

Given the variety of DMTs taken by participants, we have modest power to evaluate the association between individual DMT and suspected COVID-19 symptoms at this time. When categorizing DMTs based on mode of administration, we found no difference in the occurrence of suspected COVID-19 for injectable, oral, or infusion-based DMTs (Supplementary Table 3). Similarly, we found no differences when dichotomizing DMT as high- or standard-efficacy.

Finally, we performed a preliminary analysis of the individual DMTs among the treated participants, with the caveat of the moderate sample size at this stage (Supplementary Table 4). In this analysis, interferon-beta was associated with a lower frequency of suspected COVID-19 symptoms: of the 50 PwNID on interferon-beta, one had suspected COVID-19 (2% of the interferon-beta-treated PwNID or 1% of the PwNID with suspected COVID-19), while 49 were asymptomatic (98% of interferon-beta-treated PwNID or 9% of the asymptomatic PwNID, p=0.004). PwNID on interferon-beta are four years older on average than the rest of the PwNID (53.9+8.6 vs. 49.7+12.3, p=0.019). None of the other DMTs demonstrated a difference in the prevalence of suspected COVID-19 symptoms, including anti-CD20 therapy, which has the highest usage in the study cohort.

We gained several preliminary insights from these exploratory analyses. First, greater neurological disability is associated with a greater likelihood of suspected COVID-19 based on the CDC symptom criteria. Second, PwNID have a similar frequency of suspected COVID-19 as reference participants in our study. Third, with the caveat of small sample size and univariate analysis, interferon-beta-treated PwNID have a lower occurrence of suspected COVID-19 symptoms, while other DMT classes are not associated with higher occurrence of suspected COVID-19. While replication in larger independent samples are pending, these early results suggest that, in many MS patients, DMTs appear to be relatively safe. The interpretation of the interferon-beta finding in our study requires caution, but it is consistent with results from a recent trial in COVID-19^2^ and a case series from Italy^3^.

**Supplementary References**

1. Richardson S, Hirsch JS, Narasimhan M, et al. Presenting Characteristics, Comorbidities, and Outcomes Among 5700 Patients Hospitalized With COVID-19 in the New York City Area. JAMA 2020;323(20):2052–2059.

2. Hung IF-N, Lung K-C, Tso EY-K, et al. Triple combination of interferon beta-1b, lopinavir-ritonavir, and ribavirin in the treatment of patients admitted to hospital with COVID-19: an open-label, randomised, phase 2 trial. Lancet 2020;395(10238):1695–1704.

3. Sormani MP, De Rossi N, Schiavetti I, et al. Disease Modifying Therapies and COVID-19 Severity in Multiple Sclerosis. SSRN Journal 2020;

4. Amtmann D, Kim J, Chung H, et al. Comparing CESD-10, PHQ-9, and PROMIS depression instruments in individuals with multiple sclerosis. Rehabil Psychol 2014;59(2):220–229.

Supplementary Table 1. Multivariable Analyses of Depression and Social Support, and Suspected COVID-19 symptoms.

| Outcome | Factor | OR | 95% CI | P value |
| --- | --- | --- | --- | --- |
| Moderate to Severe Depression^a^ | NID Group^b^ | 2.22 | 1.48-3.31 | **<0.001** |
|  | Age, years | 0.97 | 0.95-0.99 | **0.003** |
|  | Female sex | 1.20 | 0.73-1.96 | 0.477 |
|  | Non-Hispanic Caucasian | 1.22 | 0.62-2.34 | 0.572 |
|  | CCI Score^c^ | 1.06 | 0.85-1.33 | 0.593 |
|  | Low Social Support | 2.28 | 1.36-3.84 | **0.002** |
| Low Social Support^d^ | NID Group | 1.82 | 1.17-2.83 | **0.008** |
|  | Age, years | 1.00 | 0.97-1.02 | 0.635 |
|  | Female sex | 0.57 | 0.36-0.91 | **0.017** |
|  | Non-Hispanic Caucasian | 0.48 | 0.26-0.88 | **0.018** |
|  | CCI Score | 1.28 | 1.04-1.55 | **0.022** |
|  | Depression^f^ | 3.01 | 1.99-4.57 | **<0.001** |
| Suspected COVID-19^g^ | Age, years | 0.98 | 0.95-1.00 | 0.094 |
|  | Female Sex | 0.85 | 0.48-1.52 | 0.587 |
|  | Non-Hispanic Caucasian | 0.63 | 0.32-1.24 | 0.182 |
|  | CCI Score | 1.03 | 0.80-1.31 | 0.832 |
|  | Depression | 1.45 | 0.91-2.31 | 0.118 |
|  | Current Smoker | 0.57 | 0.23-1.42 | 0.229 |
|  | Resident of NY, NJ or MA | 1.23 | 0.74-2.05 | 0.427 |
|  | Platform DMT^h^ | 0.78 | 0.40-1.53 | 0.398 |
|  | High Efficacy DMT^i^ | 0.80 | 0.42-1.51 | 0.419 |
|  | MSRS-R Score^j^ | 1.45 | 1.17-1.84 | **0.001** |

^a^ Moderate to Severe depression defined as PROMIS depression T-score >58.6 using established thresholds.^17^ The reference was mild depression (52.5-58.6).

^b^ Neuroinflammatory Disease

^c^ Charlson Comorbidity Index

^d^ Low social support defined as modified social support survey-5 (MSSS-5) item converted score of <40 based on 10^th^ percentile threshold. The reference was MSSS-5 score >40. . The reference was MSSS-5 score >40.

^f^ Depression defined as PROMIS depression T score > 52.5 using an established threshold.^17^ The reference was no depression

^g^ Suspected COVID-19 cases in the NID group based on CDC symptom criteria, including cough or shortness of breath OR any two of the following: fever, muscle pain, sore throat, new loss of taste or smell.

^h^ Platform DMT includes injectables and oral agents except for cladribine

^i^ High efficacy DMT includes infusions plus cladribine. The reference for treatment groups was no DMT.

^j^ Multiple Sclerosis Rating Scale revised; MSRS-R raw scores were converted to z-scores. The OR reflects a one standard deviation change in MSRS-R value.

Supplementary Table 2. Factors Associated with Suspected COVID-19 in the Entire Study Population

| Characteristic | No Symptoms (N=923) | Suspected COVID-19^a^ (N=202) | OR | 95% CI |
| --- | --- | --- | --- | --- |
| Age, Mean (SD) | 47.6 (12.3) | 45.1 (11.7) | **0.98^e^** | **0.97-0.99** |
| Male Sex, N (%) | 187 (21) | 38 (19) | 0.95 | 0.65-1.38 |
| Non-Hispanic Caucasian, N (%) | 847 (93) | 177 (88) | **0.54^e^** | **0.33-0.87** |
| PROMIS Physical Function T-score, Mean (SD) | 50.8 (12) | 49.7 (11) | 0.99 | 0.98-1.00 |
| BMI, Mean (SD)^b^ | 27.6 (7.3) | 28.2 (6.8) | 1.01 | 0.99-1.04 |
| Hypertension, N (%)^c^ | 93 (22) | 12 (14) | 0.58 | 0.30-1.14 |
| Cardiovascular Disease, N(%) | 29 (3) | 7 (4) | 1.11 | 0.48-2.56 |
| Chronic Obstructive Pulmonary Disease, N (%) | 8 (1) | 3 (2) | 1.72 | 0.45-6.56 |
| Diabetes, N (%) | 34 (4) | 13 (6) | 1.79 | 0.93-3.47 |
| Depression, N (%)^d^ | 435 (47) | 111 (55) | **1.37^e^** | **1.01-1.86** |
| Liver Disease, N (%) | 10 (1) | 6 (3) | **2.80^e^** | **1.00-7.78** |
| Peptic Ulcer Disease, N (%) | 16 (2) | 6 (3) | 1.74 | 0.67-4.49 |
| End Stage Renal Disease, N (%) | 2 (<1) | 1 (<1) | 2.29 | 0.21-25.39 |
| Connective Tissue Disease, N (%) | 20 (2) | 7 (4) | 1.62 | 0.68-3.89 |
| Cancer, N (%) | 19 (2) | 5 (3) | 1.21 | 0.45-3.27 |
| Ever Smoker, N (%) | 255 (28) | 59 (29) | 1.07 | 0.77-1.50 |
| Current Smoker, N (%) | 49 (5) | 12 (6) | 1.07 | 0.53-2.18 |

^a^ Based on CDC symptom criteria, including cough or shortness of breath OR any two of the following: fever, muscle pain, sore throat, new loss of taste or smell

^b^ Subset of 805 participants with body mass index (BMI) data

^c^ Subset of 512 patients with hypertension data

^d^ Depression defined as PROMIS Depression T score > 52.5 using an established threshold ^4^

^e^ p < 0.05

Supplementary Table 3. Factors Associated with Suspected COVID-19 in the Neuroinflammatory Group

| Characteristic | No Symptoms  N=526 | Suspected COVID-19^a^  N=104 | OR | 95% CI |
| --- | --- | --- | --- | --- |
| Age, Mean (SD) | 50.3 (12.0) | 48.8 (12.2) | 0.99 | 0.97-1.01 |
| Female Sex, N (%) | 435 (83) | 85 (82) | 0.94 | 0.54-1.62 |
| Non-Hispanic Caucasian, N (%) | 483 (92) | 90 (87) | 0.56 | 0.29-1.07 |
| Disease Duration, Mean (SD) | 18.1 (11.7) | 17.8 (11.8) | 1.00 | 0.98-1.02 |
| PROMIS Physical Function T-Score, Mean (SD) | 45.2 (11.4) | 44.6 (9.3) | 1.00 | 0.98-1.01 |
| Depression, N (%)^b^ | 247 (47) | 64 (62) | **1.81^k^** | **1.18-2.78** |
| CCI, Mean (SD)^c^ | 1.1 (1.2) | 1.0 (1.2) | 0.98 | 0.82-1.16 |
| BMI, Mean (SD)^d^ | 28.4 (7.7) | 28.9 (6.5) | 1.01 | 0.98-1.04 |
| PDDS, Mean (SD)^e^ | 2.0 (2.4) | 2.1 (1.9) | 1.01 | 0.92-1.11 |
| MSRS-R, Mean (SD)^f^ | 7 (5.4) | 9 (5.7) | **1.07^l^** | **1.03-1.11** |
| Relapse, N (%) | 33 (6) | 12 (12) | 1.95 | 0.97-3.92 |
| Urgent Care or Emergency Room Visit, N (%) | 25 (5) | 14 (14) | **3.06^k^** | **1.53-6.11** |
| Hospitalization, N (%) | 7 (1) | 2 (2) | 1.44 | 0.3.0-7.03 |
| No DMT, N (%)^g^ | 71 (14) | 16 (16) | 1.18 | 0.65-2.12 |
| DMT Mode of Administration, N (%)^h^ |  | | | |
| Self-Injectable | 103 (20) | 13 (13) | 0.59 | 0.32-1.10 |
| Oral | 87 (17) | 18 (18) | 1.06 | 0.61-1.86 |
| Infusion | 260 (50) | 54 (52) | 1.12 | 0.73-1.71 |
| DMT Efficacy, N (%) |  |  |  |  |
| High-efficacy^i^ | 259 (49) | 55 (53) | 1.18 | 0.77-1.80 |
| Standard-efficacy^j^ | 190 (36) | 31 (30) | 0.76 | 0.48-1.20 |

^a^ Based on CDC symptom criteria, including cough or shortness of breath OR any two of the following: fever, muscle pain, sore throat, new loss of taste or smell

^b^ Depression defined as PROMIS Depression T score > 52.5 using an established threshold^17^

^c^ Charlson Comorbidity Index

^d^ Subset of 461 participants with Body mass index (BMI) data

^e^ Patient determined disease step

^f^ Multiple Sclerosis Rating Scale – Revised

^g^ Disease modifying therapy

^h^ Please see Table 7 for the list of DMTs

^i^ High-efficacy DMTs include infusion agents and cladribine

^j^ Standard-efficacy DMTs include self-injectable and oral agents except for cladribine

^k^ p < 0.01

^l^ p < 0.001

Supplementary Table 4. Current Disease Modifying Therapy among the Participants with Neuroinflammatory Disorders in relation to CDC criteria for Suspected COVID-19

| DMT Name or Class, N (%)^a^ | All  N=630 | Asymptomatic  N=456 | Suspected Covid-19^b^  N=104 | OR | 95% CI |
| --- | --- | --- | --- | --- | --- |
| None | 87 (14) | 71 (14) | 16 (16) | 1.18 | 0.65-2.12 |
| Alemtuzumab | 4 (1) | 2 (<1) | 2 (2) | 5.14 | 0.72-36.89 |
| Anti-CD20: Ocrelizumab or Rituximab | 225 (36) | 190 (36) | 35 (34) | 0.91 | 0.58-1.41 |
| Cladribine | 2 (<1) | 1 (<1) | 1 (1) | 5.10 | 0.32-82.15 |
| Fingolimod | 25 (4) | 22 (4) | 3 (3) | 0.68 | 0.20-2.32 |
| Fumarates: Dimethyl Fumarate or Diroximel Fumarate | 69 (11) | 57 (11) | 12 (12) | 1.07 | 0.55-2.08 |
| Glatiramer acetate | 68 (11) | 56 (11) | 12 (12) | 1.10 | 0.57-2.12 |
| Interferon-beta | 50 (8) | 49 (9) | 1 (1) | **0.10^c^** | **0.01-0.70** |
| Natalizumab | 87 (14) | 70 (13) | 17 (16) | 1.27 | 0.72-2.27 |
| Teriflunomide | 13 (2) | 10 (2) | 3 (3) | 1.53 | 0.41-5.67 |

^a^ The reported percentage is calculated as the following: number of participants on each DMT / total number of participants in the column (all, asymptomatic, or suspected COVID-19) x 100

^b^ Suspected COVID-19 cases based on CDC symptom criteria, including cough or shortness of breath OR any two of the following: fever, muscle pain, sore throat, new loss of taste or smell.

^c^ p < 0.01
